# Analysis of Changes in Corneal Topography after 27-Gauge Transconjunctival Microincision Vitrectomy Combined with Cataract Surgery

**DOI:** 10.1155/2019/9658204

**Published:** 2019-07-10

**Authors:** Tomoyuki Watanabe, Tamaki Gekka, Akira Watanabe, Tadashi Nakano

**Affiliations:** Department of Ophthalmology, The Jikei University School of Medicine, Tokyo, Japan

## Abstract

**Purpose:**

To investigate changes in the corneal shape before and after vitrectomy, over a period of time, using a 27-gauge system.

**Methods:**

Forty-five eyes underwent a combination of cataract surgery and vitrectomy. The surgeries were performed using a 27-gauge transconjunctival vitrectomy system, in which the corneal topography could be performed up to three months after the surgery. The surgeries were performed for an epiretinal membrane in 11 eyes, a macular hole in 14 eyes, and rhegmatogenous retinal detachment in 20 eyes. All of the surgeries were performed by the same surgeon, and in all cases, a 4-port 27-gauge vitrectomy device was used. Cataract surgery in all patients was performed with a 2.4 mm corneoscleral incision at 11 o'clock. The surgeries were performed without suturing the operative wound in all cases. Corneal topography was performed using a TMS-4 topographer (Tomey Corporation, Tokyo, Japan). The examinations were performed the day before and 1 day, 1 week, 1 month, and 3 months after the surgery. The results of corneal topography for the spherical, regular astigmatic, asymmetric, and high-order irregular astigmatic components were compared before and after surgery.

**Results:**

No significant differences were seen in any of the components in the epiretinal membrane group, but significant differences were seen in the asymmetric components and the high-order irregular astigmatic components between the macular hole and rhegmatogenous retinal detachment groups (*p* < 0.05). There were no significant changes in intraocular pressure on any measurement time in the postoperative period compared to preoperative intraocular pressure.

**Conclusion:**

Irregular astigmatism was seen after surgery when 27-gauge vitrectomy with a 4-port system was performed together with cataract surgery with a 2.4 mm incision.

## 1. Introduction

Use of microincision vitrectomy surgery (MIVS) has increased since 25-gauge vitrectomy was first reported in 2002 [1]. MIVS is a form of minimally invasive surgery whereby a small incision is made above the conjunctiva and a cannula is put in place, avoiding the need to resect the conjunctiva or suture the wound. Use of 27-gauge vitrectomy that requires an even smaller incision than that needed by 25-gauge vitrectomy is now becoming widespread.

Studies comparing levels of surgical invasiveness by analysis of changes in corneal topography have reported that the changes after surgery are smaller using 25-gauge and 23-gauge MIVS than those associated with 20-gauge pars plana vitrectomy [2–5]. Furthermore, when 23-gauge MIVS and cataract surgery were performed simultaneously, there was no postoperative change in corneal topography if the operation was concluded without suturing [6]. However, those studies either did not include patients who required gas-air substitution or included only a small proportion of such patients.

The small size of the scleral wound in 27-gauge MIVS means that the procedure can be concluded without suturing, making it less invasive. However, the small diameter of the light pipe means that the amount of illumination provided is small. One method of providing supplementary lighting is chandelier illumination of the pars plana, either directly or via a fourth cannula. Using this technique, the incision size is smaller than that required with the conventional 3-port systems, but more incisions are needed.

The objective of this study was to investigate both the effect of the increased number of incisions and the effect of the disease itself on changes in corneal topography after simultaneous 27-gauge MIVS and cataract surgery using a 4-port system.

## 2. Patients and Methods

### 2.1. Patients

We retrospectively identified 45 patients (45 eyes) in whom combined cataract and vitrectomy surgery was performed using 27-gauge transconjunctival vitrectomy between December 2013 and March 2016 and in whom corneal topography was performed for up to 3 months after the surgery. The subjects comprised 34 men (34 eyes) and 11 women (11 eyes) of mean age 61.02 (range 37–77) years. The surgery was performed for an epiretinal membrane (ERM) in 11 patients (11 eyes), macular hole (MH) in 14 patients (14 eyes), and rhegmatogenous retinal detachment (RRD) in 20 patients (20 eyes).

### 2.2. Methods

Approval to conduct the study was obtained from the Ethics Committee at Jikei University School of Medicine. Informed consent was obtained from each patient. All of the surgeries were performed by the same surgeon (TG) using 4-port, 27-gauge vitrectomy and a 2.4 mm corneoscleral incision at 11 o'clock for cataract surgery.

A surgical system (Constellation or Accurus; Alcon Laboratories, Inc., Fort Worth, TX, USA) connected to an ultraspeed transformer (Dutch Ophthalmic Research Center International, Zuidland, Netherlands) was used to perform the vitrectomy. The 4 ports were created at 2 o'clock, 4 o'clock, 8 o'clock, and 10 o'clock using 27-gauge trocars (D.O.R.C. International and Alcon Laboratories). A wide-angle observation system (Resight, Carl Zeiss, Oberkochen, Germany) was used for the vitrectomy. A viscoelastic substance was used in all patients to prevent corneal damage during surgery. In all cases, the surgery was performed without suturing the scleral wound. Sulfur hexafluoride gas tamponade was used in all patients with macular hole or rhegmatogenous retinal detachment; these patients were required to remain in the prone position for 1 week postoperatively.

Corneal topography was performed using a TMS-4 topographer (Tomey Corporation, Nagoya, Japan) on the day before surgery and 1 day, 1 week, and at 1 and 3 months after surgery; Fourier analysis was also performed at the same 5 time points to extract the components for spherical power, regular astigmatism, asymmetry, and higher-order irregular astigmatism. All cases underwent intraocular pressure (IOP) measurement on the day before surgery and 1 day, 1 week, and at 1 and 3 months after surgery.

### 2.3. Statistical Analysis

The statistical analysis was performed using IBM SPSS Statistics version 2.0 (IBM Corp., Armonk, NY, USA). The spherical components, regular astigmatic components, asymmetric components, and higher-order irregular astigmatic components were analyzed using analysis of variance (ANOVA) and the Kruskal–Wallis test. Components that were not normally distributed, and for which a significant difference was found in the Kruskal–Wallis test, were then investigated using the Wilcoxon signed-rank test. The same investigations were carried out separately according to whether patients had epiretinal membrane, macular hole, or rhegmatogenous retinal detachment. The results are shown as the mean and standard deviation. A *p* value of less than 0.05 was considered to be statistically significant.

## 3. Results

### 3.1. All Patients

Table 1 summarizes the means and standard deviations (SD) of the components for spherical power, regular astigmatism, asymmetry, and higher-order irregular astigmatism. There were no statistically significant differences in the mean values for the spherical power and regular astigmatism components at any time point after surgery when compared with preoperative measurements. The mean values for asymmetric components were significant differences observed (*p* < 0.001, Kruskal–Wallis test). A comparison of the preoperative value with values obtained at 1 day, 1 week, and 1 and 3 months after surgery showed significant differences between the preoperative value and those obtained at 1 day and 1 week after surgery (*p* < 0.001, *p*=0.04, *p*=0.8, and *p*=0.189, respectively, Wilcoxon signed-rank test). The mean values for higher-order irregular astigmatism components were significant differences observed too (*p*=0.001, Kruskal–Wallis test). A comparison of the preoperative value with those obtained at 1 day, 1 week, and 1 and 3 months after surgery found significant differences between the preoperative value and those at obtained 1 day, 1 week, and 1 month after surgery (*p* < 0.001, *p* < 0.001, *p*=0.019, and *p*=0.71, respectively, Wilcoxon signed-rank test) (Table 1).

Mean IOP was 13.8 ± 2.7 mmHg before the operation, 15.0 ± 4.9 mmHg on 1 day, 14.3 ± 3.8 mmHg at 1 week, 13.9 ± 3.3 mmHg at 1 month, and 13.0 ± 3.2 mmHg at 3 months after surgery in all patients (Table 2). There were no statistically significant changes in IOP measured at any point of time in the postoperative period compared to preoperative IOP (*p*=0.108, Kruskal–Wallis test).

The following day after the surgery, IOP in one eye of the ERM patients and 2 eyes of the RRD patients was below 5 mmHg, and IOP in 2 eyes of the ERM patients and 2 eyes of the RRD patients was between 22 mmHg and 30 mmHg. In all these patients, IOP recovered to the presurgical state by 1 week postsurgery, and there was no notable variation in corneal shape.

### 3.2. Corneal Topography in Different Diseases

Tables 3–6 summarize the means and SDs of the components for spherical power, regular astigmatism, asymmetry, and higher-order irregular astigmatism for epiretinal membranes, macular holes, and rhegmatogenous retinal detachments.

#### 3.2.1. Epiretinal Membrane

There were no statistically significant differences in the mean values for the all components at any time point after surgery when compared with preoperative measurements (Tables 3–6).

Mean IOP was 13.5 ± 2.9 mmHg before the operation, 15.5 ± 6.4 mmHg on 1 day, 13.6 ± 2.8 mmHg at 1 week, 13.1 ± 3.1 mmHg at 1 month, and 12.2 ± 3.9 mmHg at 3 months after surgery in ERM (*p*=0.490, Kruskal–Wallis test) (Table 2).

#### 3.2.2. Idiopathic Macular Hole and Rhegmatogenous Retinal Detachment (Gas Tamponade Groups)

There were no statistically significant differences in the mean values for the spherical power and regular astigmatism components at any time point after surgery when compared with preoperative measurements. The mean values for asymmetric components were significant differences observed (*p* < 0.001, Kruskal–Wallis test). A comparison of the preoperative value with those on 1 day, 1 week, 1 month, and 3 months after surgery only found a significant difference between the preoperative value and that on 1 day after surgery (*p* < 0.002, *p*=0.09, *p*=0.79, and *p*=0.08, Wilcoxon signed-rank test).

The mean values for higher-order irregular astigmatic components were significant differences observed (*p*=0.001, Kruskal–Wallis test). A comparison of the preoperative value with those on 1 day, 1 week, 1 month, and 3 months after surgery only found a significant difference between the preoperative value and that on 1 day after surgery (*p*=0.009, *p*=0.10, *p*=0.06, and *p*=0.77, Wilcoxon signed-rank test) (Tables 3–6).

Mean IOP was 13.9 ± 2.6 mmHg before the operation, 14.8 ± 4.4 mmHg on 1 day, 14.5 ± 4.1 mmHg at 1 week, 14.1 ± 3.3 mmHg at 1 month, and 13.2 ± 3.0 mmHg at 3 months after surgery in MH and RRD (*p*=0.346, Kruskal–Wallis test) (Table 2).

## 4. Discussion

This study investigated the effect of the increased number of incisions as well as the effect of the disease itself on changes in corneal topography after simultaneous 27-gauge MIVS and cataract surgery using a 4-port system. Our results showed that there were no significant changes in spherical power or regular astigmatism components. However, the asymmetric components were significantly different between 1 day and 1 week after surgery when compared with the values before surgery. Higher-order irregular astigmatism components were also significantly different at 1 day, 1 week, and 1 month after surgery when compared with the preoperative value, indicating that 4-port simultaneous vitrectomy and cataract surgery using a 27-gauge system causes irregular astigmatism immediately after the procedure. Previous studies have shown that changes in corneal topography after surgery are smaller with 25-gauge and 23-gauge MIVS than with 20-gauge pars plana vitrectomy and that there is no change in corneal topography results from the day after 25-gauge vitrectomy [2–5]. Those reports suggest that the smaller the wounds, the less effect they have on corneal topography, and that the 27-gauge system, which requires even smaller wounds than those needed for 25-gauge vitrectomy, should have the least effect on changes in corneal topography. Mitsui et al. compared 25-gauge and 27-gauge vitrectomy in patients with epiretinal membrane and found no significant difference in corneal topography following [7]. However, unlike in the earlier studies, we used a 4-port system and cannot rule out the possibility that the greater number of wounds required by this system may have contributed to changes in corneal topography after surgery. Consistent with the findings of Mitsui et al., there were no significant differences for the surgically induced astigmatism in patients with epiretinal membrane, which suggests that the effect of the increased number of ports on corneal topography may be small. The factors previously reported to affect corneal shape after surgery are sutures of a wound [6], intraocular pressure [8], gas tamponade [9], etc. In this study, all the procedures performed were suture free. Moreover, variation in IOP before and after surgery showed no significant difference, showing that IOP had little involvement in the change of corneal shape.

Significant differences were only evident in patients with a gas tamponade, suggesting that the effect on corneal topography may vary depending on the disorder being treated even when the same 4-port system is used for 27-gauge vitrectomy. Park et al. have pointed out the possibility that gas tamponade may affect surgically induced astigmatism [9]. In the previous studies that found no significant difference from the day after surgery, either the subjects did not require gas tamponade or those requiring gas tamponade accounted for less than 25% of the total study population [2–5]. In our study, 75.6% of subjects had a disorder requiring gas tamponade. This was the cause of the significant differences in asymmetric components and higher-order irregular astigmatic components found in our study. Comparisons of the levels of invasiveness of the surgical procedures should be made in patients with the same disease or in those who do not require gas tamponade. In our study, there were no significant changes at 3 months after surgery in patients who had undergone 27-gauge vitrectomy with a 4-port system in whom gas tamponade was used.

## 5. Conclusion

In this study, 75% of patients who underwent 27-gauge vitrectomy with a 4-port system required gas tamponade, due to which, irregular astigmatism was apparent until 1 month after surgery but was resolved by 3 months after surgery. In the early postoperative period, no statistically significant difference was seen between the preoperative and postoperative changes in IOP between the groups, and therefore, gas tamponade affects postoperative changes in corneal topography.

## Figures and Tables

**Table 1:** Means (SDs) of all components by corneal shape Fourier analysis in all patients.

	Preoperatively	1 day postoperatively	1 week postoperatively	1 month postoperatively	1 month postoperatively
Spherical power (*p*=0.885, ANOVA)	43.63 ± 1.74 D	43.27 ± 1.89 D	43.46 ± 1.70 D	43.52 ± 1.60 D	43.56 ± 1.62 D
Regular astigmatism (*p*=0.151, Kruskal–Wallis test)	0.47 ± 0.43 D	0.51 ± 0.33 D	0.43 ± 0.27 D	0.44 ± 0.30 D	0.38 ± 0.31 D
Asymmetry (*p* < 0.001, Kruskal–Wallis test)	0.43 ± 0.32 D	0.67 ± 037 D^*∗*^ (*p* < 0.001)	0.61 ± 0.59 D^*∗*^ (*p*=0.04)	0.41 ± 0.32 D (*p*=0.80)	0.36 ± 0.23 D (*p*=0.189)
Higher-order irregular astigmatism (*p*=0.001, Kruskal–Wallis test)	0.18 ± 0.29 D	0.20 ± 0.14 D^*∗*^ (*p* < 0.001)	0.20 ± 0.18 D^*∗*^ (*p*=0.027)	0.19 ± 0.18 D^*∗*^ (*p*=0.019)	0.14 ± 0.06 D (*p*=0.71)

D: diopter; ^*∗*^postoperative compared with before surgery (Wilcoxon signed-rank test. *p* < 0.05).

**Table 2:** Means (SDs) of intraocular pressure.

	Preoperatively	1 day postoperatively	1 week postoperatively	1 month postoperatively	3 months postoperatively
All patients (*p*=0.108, Kruskal–Wallis test)	13.8 ± 2.7 mmHg	15.0 ± 4.9 mmHg	14.3 ± 3.8 mmHg	13.9 ± 3.3 mmHg	13.0 ± 3.2 mmHg
ERM (*p*=0.490, Kruskal–Wallis test)	13.5 ± 2.9 mmHg	15.5 ± 6.4 mmHg	13.6 ± 2.8 mmHg	13.1 ± 3.1 mmHg	12.2 ± 3.9 mmHg
MH + RRD (gas groups) (*p*=0.346, Kruskal–Wallis test)	13.9 ± 2.6 mmHg	14.8 ± 4.4 mmHg	14.5 ± 4.1 mmHg	14.1 ± 3.3 mmHg	13.2 ± 3.0 mmHg

ERM: epiretinal membrane; MH: macular hole; RRD: rhegmatogenous retinal detachment.

**Table 3:** Spherical equivalent component by corneal shape Fourier analysis.

	Preoperatively	1 day postoperatively	1 week postoperatively	1 month postoperatively	3 months postoperatively
ERM (*p*=0.998, ANOVA)	43.82 ± 1.49 D	43.70 ± 1.49 D	43.87 ± 1.40 D	43.85 ± 1.40 D	43.90 ± 1.40 D
MH + RRD (gas groups) (*p*=0.889, ANOVA)	43.57 ± 1.83 D	43.13 ± 2.00 D	43.33 ± 1.78 D	43.42 ± 1.66 D	43.46 ± 1.69 D

D: diopter; ERM: epiretinal membrane; MH: macular hole; RRD: rhegmatogenous retinal detachment.

**Table 4:** Regular astigmatism component by corneal shape Fourier analysis.

	Preoperatively	1 day postoperatively	1 week postoperatively	1 month postoperatively	3 months postoperatively
ERM (*p*=0.558, Kruskal–Wallis test)	0.33 ± 0.16 D	0.43 ± 0.21 D	0.34 ± 0.23 D	0.32 ± 0.15 D	0.30 ± 0.20 D
MH + RRD (gas groups) (*p*=0.328, Kruskal–Wallis test)	0.51 ± 0.47 D	0.53 ± 0.36 D	0.46 ± 0.28 D	0.48 ± 0.33 D	0.40 ± 0.34 D

D: diopter; ERM: epiretinal membrane; MH: macular hole; RRD: rhegmatogenous retinal detachment.

**Table 5:** Asymmetry component by corneal shape Fourier analysis.

	Preoperatively	1 day postoperatively	1 week postoperatively	1 month postoperatively	3 months postoperatively
ERM (*p*=0.227, Kruskal–Wallis test)	0.37 ± 0.24 D	0.56 ± 0.20 D	0.64 ± 0.74 D	0.40 ± 0.23 D	0.45 ± 0.34 D
MH + RRD (gas groups) (*p* < 0.001, Kruskal–Wallis test)	0.44 ± 0.34 D	0.71 ± 0.41 D^*∗*^ (*p* = 0.002)	0.60 ± 0.54 D (*p* = 0.09)	0.41 ± 0.35 D (*p* = 0.79)	0.32 ± 0.17 D (*p* = 0.08)

D: diopter; ERM: epiretinal membrane; MH: macular hole; RRD: rhegmatogenous retinal detachment. ^*∗*^Postoperative compared with before surgery (Wilcoxon signed-rank test. *p* < 0.05).

**Table 6:** High-order irregularity component by corneal shape Fourier analysis.

	Preoperatively	1 day postoperatively	1 week postoperatively	1 month postoperatively	3 months postoperatively
ERM (*p*=0.635, Kruskal–Wallis test)	0.12 ± 0.04 D	0.16 ± 0.09 D	0.17 ± 0.09 D	0.15 ± 0.06 D	0.14 ± 0.06 D
MH + RRD (gas groups) (*p*=0.001, Kruskal–Wallis test)	0.20 ± 0.34 D	0.21 ± 0.15 D^*∗*^ (*p*=0.009)	0.21 ± 0.21 D (*p*=0.10)	0.20 ± 0.20 D (*p*=0.06)	0.13 ± 0.06 D (*p*=0.77)

D: diopter; ERM: epiretinal membrane; MH: macular hole; RRD: rhegmatogenous retinal detachment. ^*∗*^Postoperative compared with before surgery (Wilcoxon signed-rank test. *p* < 0.05).

## Data Availability

The data used to support the findings of this study are available from the corresponding author upon request.
